# The interplay between the extracellular matrix and extracellular vesicle-associated microRNAs

**DOI:** 10.1186/s12964-025-02630-0

**Published:** 2026-01-07

**Authors:** Yunjie Wu, Nicolo Toldo, Muller Fabbri

**Affiliations:** Center for Cancer and Immunology Research, Children’s National Hospital, 111 Michigan Ave NW, Suite M5345, Washington, DC 20010 USA

**Keywords:** Extracellular matrix, Extracellular vesicles, MicroRNAs, Tumor microenvironment

## Abstract

The extracellular matrix (ECM) is a dynamic and bioactive structure that provides both physical scaffolding and regulatory cues essential for tissue homeostasis, development, and disease. In parallel, extracellular vesicles (EVs) and their associated microRNAs (miRNAs) have emerged as critical mediators of intercellular communication, influencing diverse physiological and pathological processes. Increasing evidence highlights a reciprocal interplay between the ECM and EV-miRNAs: the ECM regulates EV biogenesis, miRNA sorting, transport, and uptake, while EV-miRNAs modulate ECM composition, remodeling, and mechanobiology. This bidirectional crosstalk has profound implications for tissue repair, fibrosis, and cancer progression, where aberrant ECM-EV-miRNA signaling contributes to matrix stiffening, immune modulation, angiogenesis, and metastasis. Recent findings demonstrate that ECM mechanics and biochemical factors dictate EV cargo profiles and delivery efficiency, whereas EV-miRNAs regulate the expression of ECM proteins and remodeling enzymes, thereby shaping the microenvironment. Despite significant advances, the molecular mechanisms underlying ECM-governed miRNA trafficking and EV-miRNA-driven ECM remodeling remain incompletely understood. A deeper mechanistic understanding of ECM-EV-miRNA interactions will not only shed light on fundamental aspects of microenvironmental signaling but also open new avenues for the development of ECM- and EV-based therapies for regenerative medicine and cancer treatment.

**Graphical Abstract:**

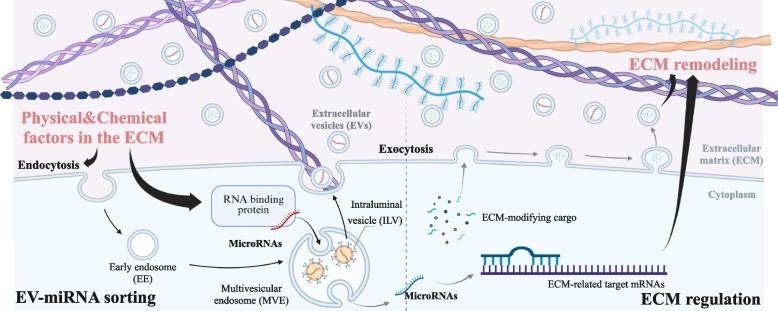

## Introduction

### Overview of the extracellular matrix

#### Biology of the extracellular matrix

The ECM is a dynamic and intricate network critical to tissue integrity and cellular function. Its complex and diverse composition is directly linked to its roles in health and disease [1]. The ECM is composed of a wide array of macromolecules collectively referred to as the matrisome. This includes over 300 secreted core proteins, such as ECM-modifying enzymes, ECM-binding growth factors, and other ECM-associated proteins, which together provide structural support and regulate cellular behavior [2]. These macromolecules are generally classified into three main groups: collagens, glycoproteins, and proteoglycans. They are synthesized within cells and assembled extracellularly into a three-dimensional, fibrous scaffold via enzymatic cross-linking. This scaffold not only provides mechanical support but also serves as a reservoir of bioactive molecules essential for cellular function [3].

The composition and structure of the ECM vary according to the anatomic location and associated function of the tissue in the body. It is broadly organized into two main forms: the basement membrane and interstitial matrix. They contain different components leading to differences in function. The basement membrane is a dense, sheet-like ECM primarily composed of type IV collagen and laminins [4]. It supports epithelial and endothelial cells by serving as a substrate for adhesion via integrins, which are heterodimeric glycoprotein receptors on the cell surface. In contrast, the interstitial matrix, found in connective tissues beneath the basement membrane, is less dense and consists of collagens, proteoglycans, and signaling molecules [5]. The specific composition and organization of ECM components in each tissue contribute to its unique mechanical and biochemical properties.

The fibrous, gel-like network of the ECM provides a dynamic microenvironment that facilitates cell adhesion, growth, migration, and signaling. This is achieved through its three-dimensional framework, cell-binding motifs, and bioactive molecules [1]. The dense collagen mesh, interspersed with nonfibrous proteins, creates tissue-specific 3D scaffolds that permit nutrient diffusion, signaling exchange, and cellular migration [6]. For example, tendon ECM is tightly packed to align with low cellularity, while submucosal ECM features more interstitial space to accommodate cell-rich environments [5]. The most abundant structural constituents of the ECM include collagen, elastin, fibronectin, and laminins. Collagens, making up approximately 30% of the ECM’s protein mass, form triple-helical structures that assemble into collagen fibers [5]. It dictates the tissue’s structural integrity, with its mechanical functionality defined by the arrangement and diversity of collagen subtypes [7]. Elastin provides elasticity to tissues such as cartilage, tendon, submucosa, and dermis, enhancing resistance to permanent deformation [7]. Fibronectin plays a crucial role in anchoring cells to the ECM and maintaining its structural integrity, while laminins are key components of the basement membrane, aiding in cell binding and ECM assembly [8]. Additionally, many ECM components expose specific molecular motifs (e.g., collagen’s GFOGER or fibronectin’s RGD sequences) recognized by integrins [9], which bridge external signals to intracellular pathways, regulating survival, differentiation, and motility [10]. Even minor ECM components, including nidogen and perlecan, stabilize the ECM by linking collagen fibers, though their roles in regulating cellular activities often overshadow their structural contributions [5]. Beyond structural functions, the ECM contains bioactive components, such as proteoglycans, growth factors and EVs. These bioactive compounds modulate key homeostatic mechanisms such as angiogenesis, ECM production, differentiation, and proliferation [2]. Proteoglycans are composed of a protein core with glycosaminoglycan (GAG) side chains. Their composition can vary depending on the tissue of origin [7]. In cartilage, they form highly hydrated gel-like networks that provide lubrication and help the tissue resist compressive forces [11]. Many growth factors are involved in cell migration, immune response, angiogenesis, and wound healing [12]. For instance, several heparin-binding growth factors have a high affinity for cell surface heparan sulfate proteoglycans [13], implicated in regulating cell behavior like differentiation, migration, and inflammation. More importantly, matrix-bound nanovesicles (MBVs), a subtype of EVs resided in the ECM, carry regulatory molecules like specific proteins, lipids, DNA and RNA that influence cellular behavior and immune regulation [14].

The ECM and resident cells engage in continuous, bidirectional communication. Cells sense and respond to mechanical, biochemical cues from the ECM through integrins and other specialized receptors [15]. Conversely, cells actively remodel the ECM by secreting enzymes like matrix metalloproteases (MMPs), which degrade matrix components and cross-linking proteins to rebuild the matrix [16]. During this ECM remodeling, bioactive fragments (e.g., growth factors, peptides, signaling molecules) are released, further influencing cellular behavior and ensures homeostasis, with ECM structure guiding cell function while cells adapt the matrix to physiological needs [17]. Collectively, the ECM is not merely a structural scaffold but a dynamic, bioactive interface that orchestrates cellular activities and tissue physiology through a complex interplay of mechanical, biochemical, and molecular signals.

#### Applications of ECM-based bio-scaffolds in tissue repair and disease

The ECM is crucial for preserving tissue structure and function, making it a vital resource for regenerative medicine. Decellularized allogeneic or xenogeneic ECM scaffolds retain structural and bioactive properties while eliminating cellular components [18]. These ECM-based bio-scaffolds including sheets, powders, and hydrogels are tailored to anatomic demands in clinical applications, including soft tissue repair, wound healing, and organ regeneration [19]. Sheets provide mechanical support and guide tissue remodeling, such as decellularized dermal sheets reinforce hernias or guide bone repair [20]. Powders and hydrogels aid chronic wound management [21] or post-infarction cardiac repair in a first-in-man trial [22], leveraging ECM-derived hydration and signaling capabilities.

Innovative strategies are broadening the utility of the ECM to ensure continued growth in regenerative medicine, including composites like ECM-coated synthetic implants, which blend biocompatibility with structural durability, and 3D-printed bioinks that replicate natural tissue architecture [23]. Notably, MBVs, isolated from decellularized matrices, offer injectable therapies that mimic parent ECM properties, showing promise in treating rheumatoid arthritis or optic nerve repair [14]. Recent advances in the characterization of endothelial MBVs further highlight their potential [24]. The study deepens our understanding of MBVs as tissue-specific ECM messengers and opens new opportunities for integrating MBV detection and functionalization into next-generation regenerative therapies. Despite challenges in balancing mechanical integrity with bioactivity, ECM scaffolds excel in recruiting host cells and fostering pro-remodeling microenvironments. The proven success of ECM bio-scaffolds in clinical settings validates their bio-functional versatility, while emerging technologies promise to unlock new frontiers in tissue repair and regenerative therapies.

### Extracellular vesicles and associated microRNAs

#### Origin and composition of EVs

Extracellular vesicles are lipid-bilayer-enclosed particles secreted by cells into the extracellular space, playing critical roles in intercellular communication. The historically described subtypes of EVs include microvesicles (MVs), exosomes, and apoptotic bodies, which are differentiated based upon their biogenesis, release pathways, size, content, and function [25]. However, according to the latest guidelines from the international Society for Extracellular Vesicles (MISEV2023), the use of these biogenesis-based terms should be reserved for cases where direct evidence demonstrates the vesicle origin, and operational definitions based on measurable properties such as size, density, surface markers and cell or tissue source are generally recommended [26].

The EV biogenesis is relatively well studied, and several modes and molecular components that drive the process have been established [27]. Traditionally, exosomes (50–150 nm) are generated by invagination of the endosomal membrane to form intraluminal vesicles within multivesicular bodies (MVBs). This process can either involve the participation of the endosomal sorting complex required for transport (ESCRT) or proceed independently of it. Detailed mechanisms including components involved in the regulation of intraluminal vesicle (ILV) biogenesis and cargo sorting are well summarized in a recent review [28]. When MVBs fuse with the plasma membrane, the intraluminal vesicles are released into the extracellular space as exosomes, a process reliant on cytoskeletal transport via microtubules and actin polymerization mediated by Arp2/3 and actin-binding cortactin [28]. In contrast, MVs (100–1000 nm) bud directly from the plasma membrane and share several proteins also involved in exosome biogenesis [27]. Beyond these two primary modes of EV biogenesis, oncosomes (1000–10000 nm) are produced through blebbing off the plasma membrane in tumor cells. These EVs are generally larger than MVs and are closely linked to cell motility [29]. Other large EVs, comparable in size to oncosomes, can also form during apoptosis. These include apoptotic bodies (100–5000 nm), which result from the shedding of the plasma membrane and the formation of spike-like microtubule protrusions known as apoptopodia [27]. Additionally, some EVs originate through mechanisms involving viral proteins. For example, retroviral-like Gag proteins can associate with the inner leaflet of the plasma membrane, bind RNA, and bud off as virus-like particles [30]. Moreover, the process of EV release can intersect with autophagy in several ways [31]. For instance, microautophagy contributes to the ILV formation, while the fusion of autophagosomes with multivesicular endosomes leads to the formation of secretory amphisomes. These structures can release diverse contents into the extracellular space, including ILVs, autophagosomal markers, and degradation products. Mature autophagosomes can also fuse directly with the plasma membrane, forming secretory autophagosomes.

These diverse mechanisms highlight the complexity of EV formation, involving multiple overlapping pathways that generate distinct EV subpopulations, carrying a broad array of bioactive molecules, with cargo composition varying according to the route of biogenesis, cell type, and physiological state. Extensive research has been conducted to characterize EV content have resulted in three major publicly available databases called ExoCarta, Vesiclepedia, and EVpedia, which catalog proteins, lipids, and nucleic acids along with associated isolation and purification methods [32]. Cargo loading appears to be highly specific to both the vesicle type and the cell of origin [32]. However, a study reported the secretion of phenotypically heterogeneous exosome populations from single cells, with no apparent correlation in the abundance of different markers on exosomes, thereby supporting the notion of stochastic sorting of membrane-associated cargo [33]. Notably, although all EVs can dynamically interact with the ECM, certain subtypes including matrix vesicles, MBVs, tissue-derived EVs (TiEVs), and exosomes secreted during migration (migrasomes), exhibit particularly close association with the ECM [34]. One study involving cargo analysis further demonstrated that MBVs constitute a distinct and unique subpopulation of EVs [35]. The influence of the ECM on EV cargo loading will be discussed in a later section.

#### EV-associated microRNAs

MicroRNAs (⁓18–30 nucleotides) are a class of small, single-stranded, non-coding RNAs (ncRNAs) that serve as key regulators of gene expression [36]. Typically, miRNAs are synthesized from DNA sequences as primary miRNA transcripts (pri-miRNAs), which are then sequentially processed into precursor miRNAs (pre-miRNAs), followed by Dicer-mediated maturation in the cytoplasm into functional, mature miRNAs. Conventionally, mature miRNAs predominantly suppress gene expression at the post-transcriptional level by binding to complementary sequences within the 3′untranslated regions (UTRs) of target messenger RNAs (mRNAs), thereby promoting mRNA degradation or translational inhibition [36]. However, emerging studies have shown that miRNAs may also interact with alternative regions such as 5′UTRs, coding regions or promoter sequences, where they may enhance the expression of the target genes under specific cellular contexts [37, 38].

Extracellular RNA (exRNA) refers to RNA molecules found outside the cells in which they were originally transcribed. These RNAs can be encapsulated within membrane-bound EVs or closely associated with proteins and/or lipid complexes [39]. The lipid bilayer structure of EVs provides protection for their RNA cargo against enzymatic degradation by extracellular RNases [32]. EVs have been shown to harbor a diverse repertoire of RNA species, including mRNA fragments, mature miRNAs, and various ncRNAs. Early studies reported that EV-associated RNAs predominantly comprised mRNA and mature miRNA sequences, with lengths peaking around 200 nucleotides but extending up to 5 kilobases [40]. Subsequent comprehensive profiling has uncovered a broader spectrum of RNA classes within EVs, including ribosomal RNAs (rRNAs), long ncRNAs (lncRNAs), PIWI-interacting RNAs (piRNAs), transfer RNAs (tRNAs), mitochondrial RNAs, Y RNAs, vault RNAs (vtRNAs) [30]. In addition, Pre-miRNAs, along with components of the RNA-induced silencing complex (RISC) like Dicer and Argonaute proteins, have also been detected in tumor-derived MVs [41]. Notably, the RNA composition of EVs does not merely reflect the bulk RNA profiles of the parent cells. Instead, accumulating evidence indicates substantial quantitative and qualitative differences between EV RNA and cellular RNA [30], suggesting a selective packaging mechanism for certain RNA species into EVs. Cellular RNA pools, known as the entire collection of intracellular RNA molecules, are dominated by full-length rRNAs and mRNAs, whereas EVs are predominantly enriched in small ncRNAs and often contain fragmented rather than intact long RNA transcripts [42]. Moreover, certain RNA species or specific sequence motifs within RNAs are packaged into EVs with different efficiencies [30], further underscoring the selectivity of this actively regulated process rather than a simple reflection of cellular RNA abundance.

Among the various species of EV-RNA, miRNAs are predominantly enriched within EVs [43]. Early studies investigating miRNA loading revealed that the abundance of specific miRNAs may differ significantly between donor cells and their secreted EVs [30], suggesting the existence of selective miRNA sorting pathways. Moreover, the miRNA composition of EVs can also be influenced by the physiological state of the originating cells. Conditions such as oxidative stress, pain, and physical exercise have been shown to modulate the levels of specific miRNAs within EVs, both in conditioned culture media from cultured cells and in the serum or plasma of patients [30]. However, several studies have reported contrasting findings. A previous study using microarray analysis found that the miRNA profiles of exosomes purified from the serum of lung carcinoma patients closely resembled those of the corresponding tumor cells [44]. Similarly, a recent small RNA sequencing study has further demonstrated that cell-type-specific miRNAs are consistently enriched both intracellularly and within EVs, indicating a degree of correspondence between intracellular and EV-associated miRNA content [45]. These discrepancies may be explained by the fact that changes in specific miRNA levels within EVs may not always be readily detectable using high-throughput approaches. These findings highlight the intricate and context-dependent mechanisms underlying miRNA sorting into EVs, which will be described in detail in subsequent sections. Collectively, the selective packaging and dysregulation of specific EV-associated miRNAs (EV-miRNAs) has been strongly implicated in the pathogenesis of various diseases, including cancer, cardiovascular disorders, and neurological conditions [36, 43].

### Interplay between the ECM and EV-miRNAs

This review explores the bidirectional interplay between the ECM and EV-miRNAs, an axis that remains underappreciated in existing literature (Summarized in Graphical abstract). The interplay between the ECM and EV-miRNAs). While the ECM significantly influences the signature and delivery of EV-miRNAs within and outside cells, EV-miRNAs contribute to the regulation of the ECM by affecting its biochemical and biophysical properties. Despite emerging recognition of this dynamic, the mechanisms underpinning ECM-mediated miRNA trafficking and miRNA-driven ECM remodeling remain poorly understood. Although previous reviews have addressed either ECM-EV communication or EV-miRNA biology, such as summarizing how EVs regulate ECM structure and remodeling, or detailed miRNA secretion mechanisms and EV-miRNA functions in disease, few integrate these processes into a unified mechanistic framework. In contrast, this manuscript synthesizes and critically evaluates emerging evidence showing how ECM biophysical cues and biochemical milieu shape selective EV-miRNA loading, transport, retention, and uptake, and how EV-miRNAs in turn drive ECM reorganization, matrix deposition or degradation, and functional remodeling in health and disease. By highlighting this two-way regulatory axis, we highlight the potential for ECM-EV-miRNA crosstalk as a key mechanism underlying tissue homeostasis and pathological remodeling. Nevertheless, several questions remain unanswered on the specific molecular pathways and the precise mechanisms through which EV-miRNAs are integrated into ECM remodeling processes. We are optimistic that ongoing research will uncover these mechanisms, leading to the development of novel therapeutic strategies aimed at targeting the ECM and EV-miRNA interplay for the treatment of ECM-associated diseases like fibrosis and cancer growth and metastasis.

## Regulation of EV-miRNA delivery by the ECM

### Packaging of EV-miRNAs regulated by the ECM

#### Selective sorting of miRNAs into EVs

During EV biogenesis, diverse RNA species, including miRNAs, are packaged into distinct EV subclasses [30]. However, the molecular mechanisms of miRNA sorting into EVs remain incompletely elucidated. Current evidence suggests that miRNA loading can occur through both passive (non-selective) and active (selective) processes [46]. Passive miRNA loading is largely influenced by the intracellular abundance of specific miRNAs and the rate of EV production, occurring without requiring their localized accumulation at sites of EV biogenesis. In contrast, active sorting involves the selective enrichment of specific miRNAs at these biogenesis sites, independent of their overall cellular abundance. This complexity of miRNA sorting is underscored by the observation that miRNA profiles in EVs often diverge significantly from those of their donor cells, implying the involvement of tightly regulated, cell type-specific mechanisms that preferentially enrich or exclude specific miRNAs from EVs [46]. A comparative profiling study of five different cell types supports this notion, demonstrating that substantial differences occur between miRNAs retained in donor cells and those secreted in EVs [47]. Depending on the cell type, 28% to 57% of expressed miRNAs exhibit preferential localization to either being selectively packaged into EVs or retained within cells. These findings highlight the substantial contribution of active sorting processes in shaping the miRNA content of EVs. Indeed, multiple factors have been implicated in regulating miRNA packaging into EVs, including miRNA abundance, subcellular localization, specific sequence motifs, post-transcriptional modifications, interactions with membrane lipids, and associations with RNA-binding proteins (RBPs).

Generally, RNAs that are small, abundant, membrane-associated, and located in the cytoplasm (rather than the nucleus) are more likely to be incorporated into EVs [30, 48]. Abundant cytoplasmic miRNAs may be passively sorted into exosomes, especially when they exceed the levels of their mRNA targets and are not bound to Argonaute 2 (Ago2) [49]. In such cases, these miRNAs are functionally released from cytoplasmic silencing roles and sequestered into exosomes. Ago2, a core component of the RISC complex, appears to modulate the selective incorporation of miRNAs into EVs [50]. Ago2 knockout leads to decreased levels of certain EV-enriched miRNAs [51], while overexpression of the KRAS oncogene has been shown to enhance the selective packaging of Ago2-associated miRNAs into EVs [52].

Specific sequence motifs also govern selective miRNA loading into EVs. A four-nucleotide motif (GGAG) is enriched in exosomal miRNAs and facilitates binding to heterogeneous nuclear ribonucleoproteins A2/B1 (hnRNPA2B1), whose SUMOylation controls this interaction and miRNAs sorting into MVBs [53]. Under oxidative stress conditions, O-GlcNAcylation of hnRNPA2B1 further promotes selective sorting of certain miRNAs into EVs [54]. Another sequence motif, called the hEXO motif, enables synaptotagmin-binding cytoplasmic RNA-interacting protein (SYNCRIP) to directly bind specific exosome-enriched miRNAs, and knockdown of SYNCRIP reduces their exosomal loading [55]. Specifically, SYNCRIP’s amino-terminal domain recognizes the hEXO motif in target EV-miRNAs, and together with its RRM domain, mediates the selective packaging of these miRNAs into exosomes, independently of their seed sequences [56]. More recently, the RNA-binding protein FMR1 has been identified as an inflammation-responsive RNA-binding protein that selectively loads miRNAs containing an AAUGC motif into exosomes through interactions with the ESCRT machinery, linking inflammasome activation to sequence-dependent miRNA sorting into EVs [57]. In contrast to RBPs that promote miRNA loading into EVs, QKI binds mature let-7b and suppresses its extracellular release, highlighting an RBP-dependent retention mechanism that shapes EV miRNA composition [58]. Interestingly, one study found that miR-1289 may mediate a novel form of post-transcriptional gene silencing mechanism by directing the transfer of mRNA into MVs via a zipcode-like sequence in the 3′UTR, rather than by inhibiting translation [59]. Furthermore, miRNA post-transcriptional modifications also influence miRNA fate. For instance, 3′uridylation of miRNAs appear to promote their incorporation into EVs, whereas 3′adenylation tends to retain miRNAs within the cell [60].

MiRNA loading into EVs is also affected by their interactions with membrane lipids. Indeed, cellular RNAs continuously interact with the cytoplasmic surface of MVBs, and their incorporation into ILVs is influenced by their affinity for raft-like regions on the outer MVB membrane [61]. In addition, one study suggests that overexpression of neutral sphingomyelinase 2 (nSMase2), a protein involved in MVB biogenesis and ceramide production, enhances extracellular miRNA levels, potentially through increased exosome production [62].

RBPs are central to RNA trafficking and sorting. Most RNAs are exported from the nucleus in complex with RBPs, forming large ribonucleoprotein particles that travel along the cytoskeleton. Mammalian cells express over 500 RBPs, and they constitute approximately 25% of EV protein content [30]. However, the distribution of RBPs across different EV subtypes and their specific roles in miRNA sorting remain insufficiently characterized. Multiple RBPs have been implicated in packaging of certain miRNAs into EVs, such as Ago2 [51, 52], hnRNPA2B1 [53, 54], SYNCRIP [55, 56], FMR1 [57], Alix [63], annexin A2 [64], major vault protein (MVP) [65], HuR [66], Y-box protein 1 [67–69],lupus La protein [70], IGF2BP1 [71], MEX3C [72], and SRSF1 [73]. Moreover, heterogeneous nuclear ribonucleoprotein K (hnRNPK) and scaffold-attachment factor B1 (SAFB) can regulate the composition of small non-coding RNAs in EVs via secretory autophagy [74]. Using TGIRT-seq, one study has discovered that EVs contain a wide array of small noncoding RNAs, such as tRNAs and Y RNAs, and that Y-box protein 1 can help these RNA sorting into exosomes alongside miRNAs [75]. Importantly, a recent review suggest that many additional miRNAs and other non-coding RNAs may also be regulated by RBPs during EV packaging, indicating a broader and more complex network of RNA–RBP interactions than currently characterized [76]. While different RBPs display binding preferences for different RNA sequence motifs, a comprehensive understanding of these interactions has yet to be established.

Additional mechanisms further illustrate the complexity of miRNA sorting. For instance, the small GTPase ARF6 directs pre-miRNAs to oncosomes alongside the miRNA processing machinery, and its activation enhances the loading of pre-miRNAs into these tumor-derived MVs [41]. MiRNA packaging into EVs is governed by a multifaceted network of passive and active processes (Summarized in Table 1). Despite growing insights into these mechanisms, the specificity with which specific miRNAs are selected for EV incorporation and the extent to which these processes differ across cell types and EV subtypes remain largely unresolved. These complexities underscore the intricate and dynamic nature of EV biogenesis and highlight the necessity for further investigation into the molecular determinants of miRNA sorting [32].

**Table 1:** Proteins involved in selective (active) miRNA sorting into EVs

Protein	Target Sequence/miRNA	Mechanism of Sorting	Reference
Argonaute 2 (Ago2)	miRNAs loaded via KRAS-MEK pathway	KRAS-MEK signaling regulates Ago2 sorting into exosomes	[52]
hnRNPA2B1	EXO-motifs (GGAG, etc.)	Sumoylation-dependent binding to specific miRNA motifs for selective exosomal loading	[53]
Caveolin-1	Stress-responsive miRNAs recognized by hnRNPA2B1	Stress-induced phosphorylation of Caveolin-1 enables hnRNPA2B1-dependent, selective miRNA loading into microvesicles	[54]
SYNCRIP (hnRNP-Q/NSAP1)	hEXO motif miRNAs	Direct binding via hEXO motif, promoting exosomal sorting	[55]
FMR1	Inflammasome-induced motif miRNAs	Binds motif-specific, inflammasome-driven miRNAs and interacts with ESCRT for stress-induced exosome loading	[57]
QKI	let-7b	QKI-mediated suppression of let-7b EV release via miRNA binding regulation	[58]
Alix	Ago2-associated miRNAs	Alix–Ago2 interaction drives miRNA packaging during EV biogenesis	[63]
Annexin A2 (ANXA2)	Sequence-independent miRNAs packaged via ANXA2	Binds miRNAs in a Ca^2+^-dependent manner and promotes their incorporation into EVs	[64]
MVP (Major Vault Protein)	miR-193a	MVP-dependent selective sorting of miR-193a into exosomes	[65]
HuR	miR-122	Stress-induced HuR binding releases miR-122 from Ago2 and promotes its selective extracellular export	[66]
YBX1 (Y-box protein 1); YBAP1/YBX1 axis	miR-223 (UCAGU motif)	YBX1 liquid–liquid phase separation (LLPS) condensates recruit specific miRNAs into exosomes; YBAP1 regulates mitochondrial availability, while YBX1 mobilizes to P-bodies then exosomes	[67–69]
La protein (Lupus La)	Selectively sorted miRNAs in high-density EVs (e.g., miR-122); Non-selective miRNAs in low-density EVs	La-mediated selective sorting into high-density EVs; Passive/non-selective sorting into low-density EVs	[70]
IGF2BP1	miRNAs loaded in IGF2BP1-dependent EVs	IGF2BP1-dependent modulation of EV cargo composition	[71]
MEX3C	miR-451a	MEX3C facilitates miR-451a loading into exosomes via AP-2/Ago2	[72]
SRSF1	miR-1246 and other exosome-enriched miRNAs sharing a common SRSF1-binding sequence motif	Binds a shared miRNA sequence motif and mediates selective enrichment of these miRNAs into pancreatic cancer exosomes	[73]

#### Influence of the physical chemical factors in the ECM on miRNA loading into EVs

EV biogenesis is closely associated with lipid membrane transport mechanisms and the physical chemical properties of the ECM that modulate this process. Lipid rafts, enriched in cholesterol and ceramide, function as precursors of MVBs [77]. Cholesterol facilitates the recruitment of the ESCRT machinery, while ceramide contributes to the induction of the negative membrane curvature necessary for ILV formation within MVBs [78]. The membrane loss that occurs during endocytosis of lipid rafts is counterbalanced by membrane gain during MVB fusion with the plasma membrane, thereby maintaining membrane homeostasis and tension [79]. Moreover, MVB fusion or exocytosis may act as a homeostatic mechanism to offset membrane loss resulting from outward budding events, such as the formation of MVs and apoptotic bodies [79]. Cells can generate traction forces and sense mechanical resistance from the ECM, so the biophysical characteristics of the ECM critically influence membrane trafficking and, consequently, EV biogenesis. Multiple studies have demonstrated that both the quantity and composition of secreted EVs are significantly affected by the mechanical properties of extracellular microenvironment [80–83]. In softer ECM environments, lipid rafts including caveolae are more readily formed, as they are not required to buffer mechanical stress [84]. Caveolae are small, cholesterol-rich, hydrophobic membrane domains characterized by the presence of the protein caveolin. Caveolin-1 plays a central role in the regulation of exosome biogenesis and cargo selection by modulating cholesterol distribution within the endosomal compartment and MVBs [85]. Through this mechanism, lipid rafts contribute to the selective incorporation of ECM components into MVBs, which are subsequently secreted via exosomes. Additionally, ECM stiffness influences cytoskeletal organization, particularly the density of the actin filament network. In cells residing on soft ECM substrates, reduced actin filament density facilitates MVB transport and fusion with the plasma membrane, thereby promoting exosome release [79]. Conversely, cells on stiff ECMs form a dense actin cytoskeleton, which serves as a physical barrier that impedes MVB trafficking and exosome secretion [82]. Of note, conflicting evidence exists regarding the impact of ECM stiffness on miRNA sorting into EVs. While one study claimed that substrate stiffness plays a vital role in the regulation of cellular miRNA expression and their sorting into EVs [83], another investigation stated that ECM stiffness primarily affects the sorting of lipids and proteins into EVs, with a relatively minor influence on miRNA content [81]. Despite this discrepancy, a study on pulmonary fibrosis demonstrated that increased ECM stiffness in fibrotic lung tissues elevates levels of pro-fibrotic miR-21-5p in EVs [86]. These observations support the notion that mechanical and biological changes within the ECM are ultimately reflected in molecular profiles of secreted EVs, including their miRNA cargo.

**Fig. 1:**
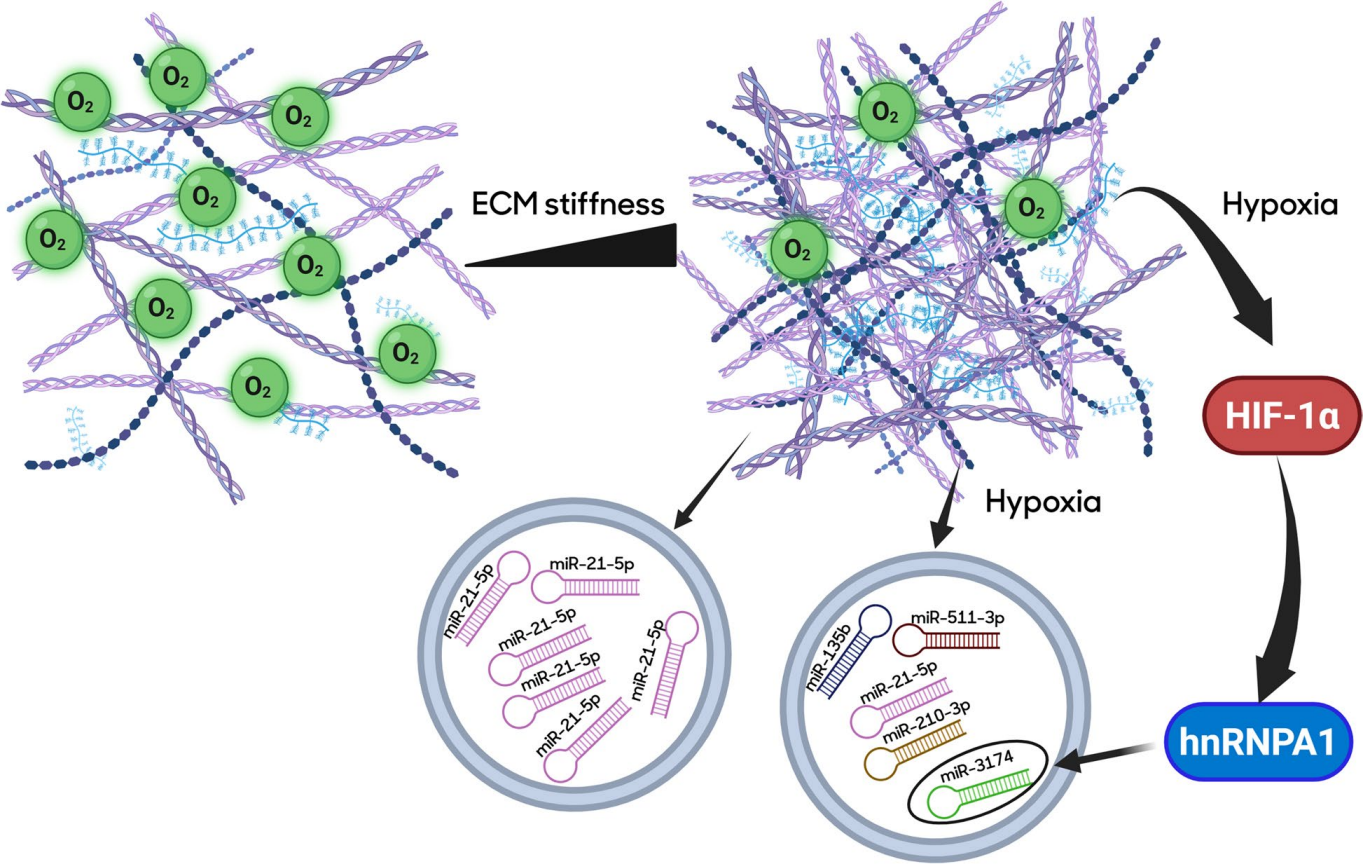
Effects of physical chemical properties of the extracellular matrix on extracellular vesicle microRNA cargo loading. Increased ECM stiffness increases miR-21-5p cargo content of EVs. Moreover, by causing hypoxia, increased ECM stiffness also promotes the EV cargo loading of the miRNAs indicated in the figure. The molecular mechanism of hypoxia-induced increased EV cargo of miR-3174 is mediated by the induction of HIF-1a which up-regulates hnRNPA1, responsible for the miR-3174 loading inside of EVs

Chemical factors in the ECM also impact EV biogenesis by modulating membrane trafficking. The ECM serves as a major reservoir of free calcium ions, which bind to lipid rafts to trigger intracellular calcium signaling and act as an essential driver in EV biogenesis, including MVB formation and fusion to the plasma membrane [79]. Soluble extracellular mediators that increase intracellular calcium, such as histamine, have been shown to enhance EV release [87]. Furthermore, pathological conditions such as cancer and tissue injury often result in increased ECM stiffness due to heightened ECM crosslinking [88], which by itself can hinder EV production [82]. These disease states are also frequently characterized by hypoxia, which has been demonstrated to promote EV biogenesis by recruiting short actin filaments to facilitate membrane trafficking [89]. Hypoxia not only increases EV secretion but also modifies EV cargo, potentially contributing to pathogenic phenotypes [90, 91]. Several miRNAs, including miR-135b [92], miR-511-3p [93], miR-21a-5p [94], miR-210-3p[95] and miR-3174 [96], have been reported to be upregulated in EVs under hypoxic conditions and to play roles in disease progression. Specifically, one study found that hypoxia-inducible factor-1α (HIF-1α) promotes transcription of miR-3174 in hepatocellular carcinoma (HCC) cells, and the RNA-binding protein hnRNPA1 facilitates its packaging into HCC-derived exosomes [96]. Figure 1summarizes the role of ECM stiffness and associated hypoxia on the loading of EV-miRNAs. Hypoxia also lowers extracellular pH as a result of increased anaerobic metabolism [97]. Acidic extracellular conditions have been shown to enhance the secretion of EVs containing caveolin-1 and to reduce membrane fluidity of EVs, which may affect their fusion and uptake properties [98]. ECM acidification is also critical for the formation of invadopodia, which are actin-rich membrane protrusions associated with ECM degradation and tumor cell invasion [99]. These structures concentrate specific membrane-bound proteases and are known to promote exosome secretion [100]. Conversely, pharmacological interventions that increase extracellular pH, such as pantoprazole (a V-ATPase inhibitor), have been shown to suppress exosome biogenesis and attenuate liver tumorigenesis [101].

The ECM might not directly affect active loading of miRNAs in EVs, but by affecting EV biogenesis and miRNA intracellular content [102], it may affect miRNA abundance in cell-derived EVs through miRNA passive EV loading mechanisms, which are largely determined by intracellular miRNA abundance and EV production rates [103, 104].

The precise mechanisms by which ECM remodeling actively or passively affects selective miRNA loading remain poorly understood. Future studies are warranted to elucidate the molecular mechanisms by which ECM-derived cues regulate the selective loading of specific miRNAs or a set of specific miRNAs into EVs under physiological and pathological conditions.

### Crosstalk between EVs and the ECM network during EV transport

#### Mechanisms of EV transport through the ECM

EVs are integral mediators of intercellular communication, facilitating the transport of biologically active molecules between cells. Like other nanoscale particles and certain viruses, EVs contribute to juxtacrine, paracrine, and endocrine signaling mechanisms [105]. Functioning as autonomous entities, EVs interact dynamically with their surrounding microenvironment, modulating the metabolic activities of neighboring cells. For instance, certain EVs act as independent metabolic units, exhibiting enzymatic activities such as asparaginase [106]. However, the efficient transport of EVs is constrained by several biological barriers, including the ECM, cellular membranes, and intravascular interfaces, which they must traverse upon release into tissue environments [107]. Normal tissues comprise a complex architecture, integrating interstitial, vascular, and cellular compartments. Connective tissues, such as those in the skin, heart, and lungs, are structured by networks of cells embedded within the ECM, which provides both mechanical stability and physical form. For EVs to reach their target cells, they must navigate the interstitial space and penetrate the ECM [108]. Key determinants of their transport include their size, shape [109], and surface properties [110], all of which influence their interactions with ECM components and their diffusion capacity. EVs may theoretically diffuse freely through interstitial fluids or interact directly with ECM molecules. Several mechanisms have been proposed to describe their movement through the ECM [111], yet our understanding remains incomplete. Elucidating these mechanisms is crucial to furthering our knowledge of EV behavior under physiological and pathological conditions [112].

The primary mechanism of EV movement through the ECM is Brownian motion, which constitutes random, thermally-driven motion [108]. ECM pore sizes vary by tissue type and can range from a few nanometers to several hundred micrometers. Given the conventional size range of EVs (approximately 30–1000 nm in diameter), most EVs are small enough to navigate through ECM pores. This size disparity supports the hypothesis that EVs can traverse interstitial spaces with minimal physical obstruction. One study has reported the presence of EVs in large quantities within interstitial spaces [113], suggesting that in many contexts, EVs neither strongly bind to ECM components nor are significantly hindered by ECM structures. In addition, EVs can also be transported via convective forces resulting from the flow of interstitial fluid in vivo [114]. Interstitial fluid within tissues is dynamic, and its flow is influenced by factors such as interstitial fluid pressure, microvascular pressure, and lymphatic activity. These factors collectively impact EV dispersion and spatial distribution. Recent findings have shown that convective transport plays a critical role in EV dissemination within laminin-rich ECM environments, while integrin-mediated interactions were observed to enhance EV retention, creating spatial concentration gradients that may facilitate targeted signaling [114].

Furthermore, EVs also actively facilitate their movement by enzymatically modifying the ECM. This process involves membrane-bound enzymes such as MMPs, aggrecanases, and heparanases [115]. MMPs, capable of degrading structural ECM components like collagen, have been detected on the surface of EVs [116]. Similarly, EVs exhibit aggrecanase activity, targeting proteoglycans such as aggrecan and heparan sulfate [117], and have been found to present heparanase on their surface [118]. Another notable example includes neutrophil-derived EVs bearing surface-bound elastase and collagenase, which can remodel the ECM directly [119]. Despite these observations, the precise molecular mechanisms through which these enzymatic processes facilitate EV mobility within the ECM remain to be fully elucidated.

#### Influence of ECM-related factors on EV retention and release within the ECM

Although EVs are generally small enough to traverse ECM pores via diffusion or convection, interactions with ECM components can hinder their mobility [110]. The ability of EVs to freely diffuse through the ECM can be modulated by several ECM-related factors.

EVs share many of their composition with their parent cell, including membrane and luminal proteins such as integrins [27]. Integrins are well known for their capacity to bind a variety of ECM components, such as fibronectin, collagen, vitronectin, and laminin [120].

Research has demonstrated that cancer-derived EVs utilize specific integrins to engage ECM components, facilitating navigation through the tumor microenvironment and enhancing metastatic potential [121]. For instance, α6β4 integrins on the EV surface exhibit high affinity for laminin [122]. Another study showed that integrin expression on EVs varies with the progression of cancer, indicating that these interactions are dynamic and context-dependent [114]. Increased integrin expression correlates with enhanced EV binding to ECM, suggesting that integrin-mediated binding is selective and dependent on ECM composition. In addition to integrins, other ECM-binding proteins are present on EVs. CD44, for example, enables EVs to bind hyaluronic acid [122], while surface-bound fibronectin promotes interactions with heparan sulfate [123]. Moreover, the ECM itself can sequester various signaling molecules, including growth factors [124], thereby forming concentration gradients essential for localized signaling [125]. This reservoir function of the ECM influences the spatial distribution and activity of growth factors [126] and, by extension, EVs carrying associated receptors or ligands. Several growth factor receptors (e.g. EGFR [127], FGFR1 [128]) and ligands such as TGF-β [129] have been identified on EV surfaces, contributing to their retention within the ECM.

Beyond specific ligand-receptor interactions, non-specific interactions including electrostatic forces also influence EV dynamics within the ECM [130]. Under physiological conditions, EVs typically possess a net negative surface charge, attributed to their membrane lipids and surface proteins, such as glycoproteins [131]. This negative charge induces repulsive forces among EVs, promoting their dispersion and preventing aggregation. However, various physiochemical factors such as pH and ionic strength significantly modulate surface charge of EVs. Increased ionic conductivity in the ECM can compress the electrical double layer surrounding EVs, reducing repulsive forces and promoting aggregation [132]. Similarly, acidic conditions can increase EV surface negativity and enhance their tendency to cluster [132]. Since the ECM itself carries a substantial net negative charge owing to its abundance of glycosaminoglycans and proteoglycans [2], electrostatic attraction between EVs and the ECM is considered unlikely. Further research is required to fully elucidate the extent and significance of these electrostatic interactions in EV retention.

A recent study demonstrated that the mechanical properties of the ECM, along with a specific mechanism involving EV deformability, influence EV diffusion, even though EVs are larger than the pore size of the ECM [133]. This mode of transport distinct from passive, free diffusion. Notably, EVs were found to diffuse more efficiently in rigid, stress-relaxing ECMs compared to flexible, stress-relaxing matrices. This phenomenon is likely attributable to the fact that stiffer ECMs facilitate more rapid interstitial fluid flow, thereby promoting EV transport. These findings highlight the critical role of ECM biomechanics in regulating EV mobility.Taken together, these interactions between EVs and the ECM contribute to both the retention and release of EVs within tissues (Summarized in Table 2). By serving as a dynamic reservoir, the ECM regulates the local availability of EVs and facilitates the transfer of bioactive molecules, including a variety of EV-miRNAs, to target cells. Consequently, the ECM is a critical modulator of EV distribution, transport, and functional outcomes in tissue microenvironments.

**Table 2:** Receptor-ligand and non-specific interactions affecting EV transport through ECM

EV cargo/surface property	ECM components/factors	Reference
Laminin-binding integrins (α3β1, α6β1) and general EV size/adhesion characteristics	Laminin-rich ECM and interstitial flow (convection)	[114]
EV-associated matrix remodeling enzymes (MMPs, heparanase, hyaluronidase, and aggrecanases) and their regulators (e.g., EMMPRIN, TIMPs)	Collagens, proteoglycans, elastin, fibronectin, and glycosaminoglycans (e.g., hyaluronan, heparan sulfate)	[115]
EV-associated MMPs	ECM proteins (collagens, proteoglycans, elastin, fibronectin)	[116]
Aggrecanases/ADAMTS	Aggrecan	[117]
Heparanase	Heparan sulfate	[118]
Surface-bound neutrophil elastase (NE) and integrin Mac-1 (CD11b/CD18)	Type I collagen, elastin, and other ECM structural proteins in lung interstitium	[119]
Integrins	Fibronectin, collagen, laminin, ICAM/VCAM motifs, etc	[120]
Integrins (e.g., α6β4, α6β1, αvβ5)	Organ-specific ECM niches (e.g., laminin-rich lung ECM, fibronectin-rich liver ECM)	[121]
Exosomal adhesion molecules (high CD44 and α6β4 integrin) and proteases (uPAR, MMP2, MMP3, MMP9, MMP14, ADAM17, ADAMTS1/8)	Hyaluronic acid, collagens (I, II, IV), laminins (LN111, LN332), fibronectin	[122]
Fibronectin	Heparan sulfate proteoglycans	[123]
Fibronectin growth factor–binding domains	Growth factors (e.g., PDGF, VEGF, FGF families, TGF-β) bound to fibronectin domains	[126]
FGFR1 as a receptor on the EV surface	FGF ligands	[128]
EV-associated TGF-β pathway components (e.g., TGF-β ligand, receptors/co-receptors, miRNAs/lncRNAs modulating TGF-β signaling)	ECM-deposited latent TGF-β and associated ECM modulators	[129]
EV surface molecular repertoire	ECM molecules and extracellular milieu factors (e.g., heparan sulphate proteoglycans, cytokines, growth factors, albumin, complement factors)	[131]
EV surface physicochemical properties	Colloidal environment factors (ionic strength, pH, multivalent ions)	[132]
Aquaporin-1	ECM mechanical properties (stiffness, stress-relaxation, polymer mesh size)	[133]

### Functional transfer of EV-miRNAs regulated by the ECM

#### EV uptake and EV-miRNA release into recipient cells

Following their traversal of biological barriers, EVs can modulate recipient cell behavior either by interacting with cell surface receptors to trigger downstream signaling cascades without internalization or through cellular uptake via multiple internalization pathways [31]. The mode of EV interaction with and/or entry into cells ultimately determines their functional effects [32]. For EVs to effectively deliver EV-miRNAs, cellular uptake is typically required. This uptake can occur via both selective and non-selective processes, influenced by multiple factors including the physiological state and identity of the recipient cell, as well as the molecular composition of the EV surface, particularly its proteins, lipids, glycans, and net negative charge [134]. For instance, integrin expression on tumor-derived EVs has been shown to mediate tissue-specific tropism, facilitating the establishment of pre-metastatic niches in distant organs [121].

The uptake of EVs occurs in several ways include clathrin/caveolin-mediated endocytosis, macropinocytosis, phagocytosis, lipid raft-mediated internalization, and direct membrane fusion [135]. Among these, endocytic routes have been studied most extensively, partly due to the technical difficulty of detecting membrane fusion events. However, the factors that govern the specificity of EV interactions with recipient cells are still not fully understood. It remains unclear whether cells actively discriminate between different EV subtypes or modulate uptake based on their physiological condition. While mechanisms of EV internalization are relatively well-defined, the intracellular delivery and functional integration of intra-vesicular cargos, particularly miRNAs, require further elucidation. Three primary mechanisms have been proposed for the transfer of miRNAs from EVs to recipient cells [136]. Firstly, EVs can deliver miRNAs through well-characterized endocytic pathways followed by cargo release to the endosome [137]. Secondly, EV-miRNA is transferred between T and B cells via synapse formation in both EV and cell-contact dependent manner, a process inhibited by neutral sphingomyelinase inhibition or Rab27a knockdown [138]. Interestingly, an alternative, non-vesicular mechanism of RNA delivery via tunneling nanotubes has also been reported, particularly for mRNA, further broadening the scope of intercellular RNA transfer mechanisms [139]. Thirdly, direct fusion between the EV and plasma membrane contributes to the cytoplasmic release of miRNA cargo [140]. Once internalized, miRNAs may become functionally active through release into the cytoplasm or within the endosomal compartment, where they potentially modulate signaling pathways [141]. However, not all EV-derived miRNAs from donor cells exert functional effects within recipient cells. Evidence suggests that miRNA packaging into EVs may also serve a homeostatic function in donor cells, acting as a quality control mechanism to eliminate unwanted or mislocated miRNAs [142]. This raises the possibility that some EV-miRNAs may primarily serve intracellular housekeeping roles rather than intercellular communication. Additionally, EVs may remain attached to recipient cell surfaces without internalization, potentially participating in processes such as antigen presentation or surface-mediated signaling, without contributing to functional transfer of EV-miRNAs [143].

For EV-derived miRNAs to exert functional effects, they must ultimately reach the cytoplasm of recipient cells. If EV uptake occurs via the endosomal pathway rather than through direct, non-selective fusion of vesicular and cellular membranes, the efficiency of EV-miRNA delivery becomes a crucial determinant for their therapeutic or biological effect. Despite growing evidence for EV-mediated miRNA transfer, its functional significance remains contested, largely because of the low concentration of miRNAs within EVs [144]. Quantitative studies have demonstrated that even for relatively abundant miRNAs, more than 100 EVs may be required to deliver a single copy of the miRNA to a recipient cell [145]. This low stoichiometry has led to skepticism regarding the capacity of EVs in miRNA-mediated regulation of the recipient cell. Nevertheless, it is plausible that rare, miRNA-enriched EVs could be selectively taken up by target cells, leading to notable silencing of mRNA targets, or the accumulation of numerous EVs containing low miRNA levels could collectively achieve functionally relevant intracellular concentrations [146]. After EV internalization into the endosome, miRNAs can be released either through degradation of the EV membrane within the endosomal lumen or via direct fusion of the EV membrane with the endosomal membrane, a process known as endosomal escape [30]. Breach of the EV lipid bilayer may expose miRNA cargo within the endosome. This exposure can activate intracellular pattern recognition receptors, such as Toll-like receptors (TLRs), which recognize single-stranded RNA within endosomal compartments. For example, lung cancer cells release EVs enriched in miR-21 and miR-29a, which are taken up by surrounding immune cells [141]. Once internalized, these miRNAs activate endosomal TLR8 signaling, driving a pro-inflammatory phenotype that supports tumor progression. Similarly, exosomal miR-21 and miR-155 have been shown to mediate the cross-talk between neuroblastoma cells and human monocytes, thereby promoting resistance to chemotherapy [147]. In addition to EV disruption within the endosome, inefficiencies in endosomal escape remains a limiting barrier to the functional delivery of EV-miRNAs into the cytoplasm [148]. If EVs fail to escape the endosomal compartment, they are likely to be degraded by fusion with lysosomes, hindering the functional delivery of their cargo. Although certain EVs may possess fusogenic properties that promote endosomal escape to avoid the degradative pathway [30], the molecular mechanisms underpinning this process remain poorly defined. Moreover, EV uptake via endocytosis and engulfment pathways such as phagocytosis and macropinocytosis complicates the cytoplasmic delivery of functional miRNAs, as these routes primarily direct cargo to lysosomes for degradation [149].

Despite these challenges, EVs are capable of delivering both surface-bound and intraluminal contents into the cytoplasm through fusogenic processes [31]. Several studies have demonstrated that EV-miRNAs can modulate measurable changes in gene expression in recipient cells once successfully delivered and protected from degradation under experimental conditions [30]. However, it remains difficult to definitively attribute these effects to EV-mediated delivery rather than endogenous upregulation of miRNAs in response to EV exposure or co-culture conditions [146]. A deeper understanding of uptake specificity, endosomal escape, and intracellular routing of EVs is essential for fully elucidating biological and therapeutic potential of EV-miRNAs.

#### Influence of the ECM on EV uptake and EV-miRNA cytosolic delivery

The ECM provides a highly dynamic and complex microenvironment that critically modulates EV uptake and cytosolic delivery of EV-miRNAs. Numerous studies have demonstrated that ECM-related factors including mechanical properties, molecular components and the environmental conditions they provide, affect EV interactions with recipient cells and subsequent intracellular processing of their contents.

One key mechanism through which the ECM exerts influence of EV internalization into target cells is via the selective retention and release of EVs. For instance, EVs derived from HEK293T cells exhibit preferential uptake by the same cell type [150], suggesting that the degree of selectivity and specificity of EV uptake depends on the preferential tropism of EVs for certain cell types [31]. However, other reports suggest that natural EVs were taken up by any cell type in a non-selective way, regardless of EV origin [135], indicating that such specificity may be context-dependent. Notably, filopodia-recruited EVs within endosomes have been observed to be transported to the endoplasmic reticulum before being sorted into lysosomes for degradation, suggesting a targeted pathway for efficient cytosolic delivery of EV-miRNA cargo into the translational machinery [151]. Filopodia are cellular protrusions formed by actin filaments that constantly engage in the environmental sensing, and they explore the ECM and sense the stiffness of the environment [152]. ECM stiffness has been reported to influence EV secretion and uptake behaviors [81, 153].Furthermore, ECM stiffness may indirectly modulate cytosolic delivery of EV content, by affecting endosomal escape of EVs, a crucial step for functional delivery of EV-miRNAs. Changes in ECM stiffness can alter cell morphology and actin cytoskeleton dynamics [154], which in turn may influence the efficiency of EV escape from endosomal compartments into the cytosol. Supporting this, one study show that ECM stiffness regulates endosomal escape rates in the context of bacterial uptake [155], which may analogously apply to EV cargo delivery.

Beyond mechanical characteristics, specific ECM components are critical modulators of EV behavior. The ECM and pericellular matrix can store EVs, affecting their mobility and availability to target cells through potentially selective interactions. For instance, heparan sulfate proteoglycans that are a part of the ECM on the recipient cell surface are required for efficient uptake of tumor-derived EVs via endocytosis and subsequent release of EV content for their functional activity [156, 157], with blocking by heparin reduces the internalization of EVs in cell culture [158]. Fibronectin as another ECM component could simultaneously bind to heparan sulfate proteoglycans on the EVs and plasma membranes, facilitating cellular uptake of EVs [123]. Additionally, fibronectin alters the expression of connexin 43 in alveolar epithelial cells [159]. Connexin 43 is the most widely expressed gap junction protein, which is also present in EVs to modulate the interaction and transfer of information including miRNAs between EVs and acceptor cells [160]. Through this mechanism, fibronectin may indirectly modulate cytosolic delivery of EV-miRNAs via cell-EVs interaction. Integrins are key cellular receptors for various ECM constituents, further contributing to selective EV uptake. Specific integrins have been shown to mediate preferential internalization of EVs in certain primary tumors [161]. These interactions collectively underscore the role of ECM composition in guiding EV-cell recognition, uptake, and downstream functional delivery of EV cargos.

Environmental conditions also influence EV internalization into target cells. EV uptake is highly temperature-dependent, indicating that the internalization process is primarily driven by active, energy-dependent mechanisms rather than passive diffusion [162]. Recipient cells incubated at 4 °C exhibit a significantly reduced capacity to internalize EVs across multiple cell types compared to those incubated at 37 °C [162–164]. One of these studies revealed that EV uptake occurs predominantly through clathrin-independent endocytosis and macropinocytosis, and requires cholesterol, highlighting the minimal role of clathrin-dependent pathways and the critical importance of lipid raft integrity [162]. Notably, macropinocytosis facilitates higher cargo delivery efficiency of EVs to pancreatic cancer cells compared to liposomes, emphasizing its potential importance as a productive pathway for cytosolic delivery of EV-miRNAs [165].

Other microenvironmental factors, such as acidic pH, hypoxia, have also been shown to regulate EV uptake by recipient cells. Acidic pH conditions promote substantial intra-tumoral uptake of exosomes. Notably, exposure to exosomes generated in an acidic environment induces migratory and invasive abilities in pH-naïve melanoma cells, likely through the transfer of metastatic exosomal proteins [166]. Furthermore, exosome uptake via membrane fusion is significantly increased in melanoma cells cultured under low pH conditions, thereby facilitating their cargo delivery [98]. In addition, hypoxic conditions similarly augment EV uptake. Endothelial cells exposed to hypoxia demonstrate a greater uptake of EVs relative to those maintained under normoxic conditions [167]. Mechanistically, this hypoxia-induced enhancement of EV uptake is dependent on increased endocytosis of heparan sulfate proteoglycans, which predominantly occurs through the lipid raft-mediated endocytic pathway [168]. Indeed, several studies demonstrated that EVs produced under hypoxic conditions are enriched in specific miRNAs, and their uptake by target cells results in more efficient delivery of these miRNAs [92–96]. These observations indicate that EV uptake is highly responsive to the microenvironmental context, which can either promote or inhibit the functional delivery of EV contents, especially EV-miRNAs.

These findings underscore the complex and dynamic interplay among EVs, the ECM, and recipient cells (Summarized in Table 3). A comprehensive understanding of these interactions is critical, as they offer promising avenues for the development of targeted therapeutic strategies and highlight the substantial potential of this field for future research and clinical translation.

**Table 3:** The effect of ECM/environmental factors on EV uptake and EV-cargo delivery

ECM-related factor	Mechanism of interaction	Effect on EV uptake	Effect on EV cargo delivery	Reference
ECM stiffness (soft vs stiff substrates)	Modulates cargo sorting into MSC-EVs, altering EV composition	Stiff-derived EVs enhanced macrophage uptake compared with soft-EVs	Altered protein/lipid loading influences delivery behavior; miRNA changes minor	[81]
Fibrotic ECM	ECM stiffness–dependent mechanotransduction reprograms fibrocyte miRNA expression	Not directly assessed	Enhanced EV-mediated delivery of pro-fibrotic miRNA (miR-21-5p)	[86]
Hypoxic TME	Increases exosome secretion	Elevated exosome uptake by endothelial cells	Functional delivery of exosomal miR-135b	[92]
Hypoxic TME	Alters EV biogenesis and enhances EV production	Increased EV uptake by target cells	Functional delivery of EV-miR-511-3p targeting TRAF6/S1P/NF-κB axis	[93]
Hypoxic neuronal microenvironment	Rapidly increases neuronal EV secretion	Neuron-derived EVs are robustly internalized by phagocytic cells	Functional enrichment and transfer of miR-21a-5p	[94]
Hypoxic TME	Increases neuroblastoma EV secretion	Enhanced uptake by target neuroblastoma cells	Functional delivery of exosomal miR-210-3p	[95]
Hypoxic TME	Increases exosome production	Increased uptake by Human umbilical vein endothelial cell (HUVECs)	Functional delivery of exosomal miR-3174 to HUVECs inhibits HIPK3/p53 and HIPK3/Fas	[96]
Acidic pH in tumor microenvironment (TME)	Alters exosome membrane lipid composition (rigidity, SM/GM3 enrichment) and promotes direct membrane fusion efficiency	Increased exosome uptake by melanoma cells under acidic conditions	Enhanced delivery of exosomal proteins (e.g., caveolin-1); specific miRNA delivery not assessed	[98]
Fibronectin	Fibronectin binds heparan sulfate to mediate exosome-cell interaction	Enhanced exosome-cell interaction/uptake via fibronectin-HS linkage	Facilitated delivery of exosomal cargo via fibronectin-mediated uptake	[123]
Heparan sulfate proteoglycans (HSPGs)	HSPG-mediated binding and internalization of EVs	Rapid, dose-dependent, and HSPG-dependent EV uptake by HEK293T	Not assessed	[150]
Filopodia-rich cell surface regions	Filopodia-mediated EV surfing and actin-dependent endocytosis	Directed EV transport along filopodia to endocytic hot spots	Intracellular trafficking via endosomes/ER may enhance cargo delivery	[151]
Stiff ECM	Akt-Rabin8-Rab8 mechanotransduction pathway activated by stiff ECM	Increased exosome secretion	Enhanced delivery of EV cargo (e.g., Jagged1)	[153]
ECM nanostructure and dimensionality	ECM topography–dependent actin cytoskeleton remodeling via integrin-mediated mechanosensing	Not assessed	Not assessed	[154]
HSPGs	HSPG-dependent endocytosis	Inhibited by heparin or HS degradation	Functional delivery of exosomal cargo	[157]
Heparin	Competes with HSPGs, inhibiting EV binding and internalization	Significantly reduces EV internalization	miRNA or other EV cargo delivery inhibited indirectly	[158]
Fibronectin	Regulates connexin43 (Cx43) expression via integrin signaling; Cx43 gap junctions mediate EV docking and direct cargo transfer	Cx43-dependent gap junction-mediated EV uptake	Functional EV cargo delivery (e.g., small RNAs) enabled by Cx43	[159, 160]
Integrins	Mediate EV binding to specific ECM components or cell-surface receptors	Not assessed	Not assessed	[161]
Temperature	Internalization is active, energy-dependent; inhibited at low temperature	EV uptake significantly reduced at 4 °C compared to 37 °C	Reduced delivery of EV cargo into recipient cells at low temperature	[163]
Acidic TME	Acidic TME may promote matrix remodeling	Enhance intra-tumoral uptake of exosomes	Enhanced transfer of pro-invasive metastatic molecules via exosome (proteins); specific miRNA delivery not assessed	[166]
Hypoxic TME	Enhances proteoglycan-dependent endocytosis (increased HSPG/lipid raft pathway)	Increased hypoxia-sensitive, HSPG-dependent EV internalization	Enhanced delivery of EV cargo fueling lipid droplet phenotype	[168]

## Role of EV-miRNAs in ECM regulation during development and disease

### Functional impacts of EV-miRNAs in health and disease

EV-miRNAs have emerged as critical mediators of intercellular communication, exerting broad regulatory effects on diverse biological processes. Accumulating evidence from both in vitro and in vivo studies highlights the functional significance of EV-miRNAs across various physiological and pathological contexts by modulating gene expression, influencing immune cell behavior, and serving as promising candidates for diagnostic biomarkers and therapeutic agents [169].

In the context of tissue repair, EV-miRNAs derived from mesenchymal stem cells (MSCs) have demonstrated notable contribution to skin regeneration and rejuvenation by targeting genes involved in inflammation, cell migration, proliferation, and apoptosis [170]. In the cardiovascular system, EV-miRNAs are widely involved in the initiation and progression of various cardiovascular diseases [171]. A recent study also show that cardiac tissue-resident vesicles can differentially modulate anti-fibrotic signaling in an age- and sex-dependent manner through synergistic miRNA effects [172]. These vesicles exhibit distinct miRNA cargo compositions associated with aging-related ECM alterations and hormonal differences, thereby influencing fibroblast activation, collagen deposition, and overall ECM turnover. In central nervous system (CNS) disorders, specific EV-miRNAs have been identified not only as potential diagnostic biomarkers but also as key pathological regulators and therapeutic agents [173]. In metabolic diseases such as diabetes mellitus, EV-miRNAs are increasingly recognized for their involvement in disease progression, primarily influencing pancreatic β-cell injury and insulin resistance [174].

More importantly, EV-miRNAs have been extensively studied in cancer biology, where they are involved in multiple stages of tumor development [175]. Within the tumor microenvironment, EV-miRNAs can promote tumorigenesis by enhancing cell proliferation and metastasis through facilitation of angiogenesis, modulating immune evasion, and remodeling of the ECM [176, 177].

### EV-miRNA-mediated regulation of the ECM in homeostasis and development

#### Regulation of ECM composition and structure by EV-miRNAs

EVs play an integral role in the evolution of the ECM in both physiological and pathological states. They have been shown to influence the composition and structural organization of the ECM both directly and indirectly [109]. EVs can physically integrate into the ECM as bioactive components, contributing to its architecture and mechanical properties. Additionally, they possess surface-associated enzymes and molecular contents that actively participate in ECM remodeling [34]. For instance, exosomes derived from human adipose-derived MSCs have been demonstrated to inhibit gene and protein expression of key ECM components such as collagen I, collagen III, fibronectin, and α-smooth muscle actin in keloid fibroblasts, effectively suppressing ECM production in keloids [178]. Conversely, MSCs-derived EVs can promote fibroblast proliferation and enhance the synthesis of type I and III collagens and elastin in a dose-dependent manner [179]. Supporting this, stimulation of fibroblasts with adipose tissue-derived stem cell exosomes increases both mRNA and protein levels of collagen I, collagen III, MMP1, bFGF, and TGF-β1 [180]. Notably, EVs modulate ECM composition indirectly, predominantly via the delivery of miRNAs that regulate cellular signaling pathways. These miRNAs function by binding to complementary mRNA targets, leading to their degradation or translational repression, thereby controlling gene expression related to ECM synthesis and remodeling. Recent work has demonstrated that MBVs derived from cartilage versus small intestinal submucosa (SIS) exhibit distinct tissue-specific miRNA signatures, with cartilage-derived MBVs enriched in anti-angiogenic miRNAs (e.g., miR-140-3p, miR-455-5p, miR-148a-5p) and SIS-derived MBVs enriched in pro-angiogenic miRNAs (e.g., miR-143-3p, miR-181a, miR-21-5p) [181]. These findings confirm that MBVs act as functional messengers of ECM-encoded signals, and that their miRNA cargo is selectively shaped by the biochemical and structural properties of the parent ECM, highlighting a direct link between ECM composition and EV-mediated regulation of angiogenesis and matrix remodeling. Comprehensive profiling of miRNAs within EVs has been compiled in the EV-miRNA database, which catalogs over 1,000 miRNAs and identifies their association with specific cellular origins and disease contexts [182].

Many miRNAs present in EVs suppress matrix synthesis by targeting mRNAs encoding ECM components or regulators. For example, human amniotic fluid stem cell-derived exosomes contain miRNAs such as let-7-5p, miR-22-3p, miR-27a-3p, miR-21-5p, and miR-23a-3p, which inhibit the TGF-β signaling pathway by targeting TGF-β receptor type I and type II (TGFBR1, TGFBR2) [183]. Consistently, human bone marrow mesenchymal stem cell-derived EVs transfer let-7a-5p to inhibit TGF-β-induced fibroblast activation and collagen secretion in vitro and reduce capsular fibrosis in vivo [184]. Umbilical cord blood mesenchymal stem cell-derived exosomes are enriched in miR-21-5p and miR-125b-5p, which target TGFBR2 and TGFBR1, respectively, suppressing myofibroblast formation and collagen I deposition [185]. Human bronchial epithelial cell-derived EVs carry miRNAs (miR-16, miR-26a/b, miR-141, miR-148a, miR-200a) that attenuate myofibroblast differentiation and cellular senescence by inhibiting both canonical and non-canonical WNT signaling pathways, thus interfering with TGF-β–WNT crosstalk [186]. Similarly, macrophage-derived EVs under high-glucose conditions carry miR-17-5p, which targets TGF-β receptor II to attenuate osteogenic differentiation of vascular smooth muscle cells, highlighting another EV-mediated mechanism for suppressing pathological ECM [187]. In addition, human umbilical cord mesenchymal stem cell-derived EVs are enriched in miR-148a-3p, which targets Hsp90b1 to suppress fibroblast collagen I, fibronectin, and α-SMA synthesis and secretion in silica-induced pulmonary fibrosis, thereby attenuating ECM accumulation [188]. Hepatocyte-derived EVs contain miR-423-5p, which suppresses the Cola1α, α-SMA, Mgll, and FAM3 expression in hepatic stellate cells [189]. Induced pluripotent stem cell-derived MSC exosomes contain miR-432-5p that inhibits translocation-associated membrane protein 2, a key modulator of collagen biosynthesis in corneal stromal stem cells, preventing excessive ECM deposition [190]. Myogenic progenitor cell-derived exosomes deliver miR-206, which represses Rrbp1, a master regulator of collagen biosynthesis, thus preventing fibrosis [191]. Adipose MSC-derived EVs enriched with miR-192-5p target IL-17RA, downregulating p-Smad2/p-Smad3 signaling and profibrotic proteins including collagen [192]. Pulmonary vascular endothelial cell-derived EVs carry miR-107 that targets hypoxia-inducible factor-1α, suppressing fibroblast-mediated fibrosis [193].

Conversely, miRNA-mediated ECM enhancement occurs through targeting mRNAs that promote ECM degradation directly and indirectly. MMPs are responsible for ECM breakdown and are implicated in degenerative diseases such as intervertebral disc degeneration and arthritis. MSC-derived EVs carry miRNAs that suppress MMP expression, thereby decreasing ECM degradation. Examples include miR-150-5p targeting of MMP14 and VEGF [194], miR-532–5p targeting of RASSF5 [195], and miR-202-3p targeting of MMP11 [196]. Nucleus pulposus cell-derived exosomes transfer miR-15a to nucleus pulposus MSCs, promoting chondrogenic differentiation by downregulating MMP3 expression [197]. Additionally, EVs from chondrogenic bone marrow MSCs carry miR-205-5p, which targets MDM2, reducing proinflammatory cytokines, MMPs, MAPK, and NF-κB pathways, ultimately protecting joint integrity [198]. MSC-derived EVs delivering miR-125a-5p downregulate MMP13 by targeting E2F2, while simultaneously enhancing expression of collagen II, aggrecan, and SOX9, which support ECM formation [199]. In addition to MMP targeting, EV-associated miRNAs promote ECM synthesis and deposition. For example, miR-155-5p enriched in EVs inhibits the Runx2 gene, whose overexpression impairs ECM secretion. Treatment with exosomes carrying miR-155-5p enhances ECM secretion, cellular proliferation, and migration in chondrocytes, thereby attenuating osteoarthritis progression [200]. Fibroblast-derived EVs containing miR-199a-5p target caveolin-1, promoting skeletal muscle fibrosis by increasing α-SMA, collagen, and fibronectin expression [201].

#### Regulation of ECM remodeling by EV-miRNAs during tissue development

During tissue development, cellular communication is essential for tissue morphogenesis. This communication is mediated through junctions, receptors, and soluble factors, facilitating the dynamic interaction between cells and their microenvironment [202]. Such bidirectional signaling orchestrates organogenesis and promotes the spatial and functional organization of cells into structured tissues. The ECM further contributes to organogenesis by regulating several processes, including cell adhesion, proliferation, migration, survival, and differentiation.

A range of EV-miRNAs has been shown to play a regulatory role in development by modulating specific ECM components. For instance, fibronectin, a glycoprotein crucial for embryogenesis, functions by binding integrins to mediate cell adhesion, proliferation and tissue development [202]. miR-17-5p, found in various types of EVs, impairs tissue development by suppressing fibronectin and fibronectin type III domain containing 3A (FNDC3A)expression [203]. Versican, a chondroitin sulfate proteoglycan abundant in the ECM of the cardiovascular system, also undergoes miRNA-mediated regulation during development. MiR-138 plays a role in cardiac patterning [204], whereas miR-143, upregulated by myocardin, downregulates versican in differentiating smooth muscle cells, thereby inhibiting cell migration [205]. Notably, both miR-138-5p and miR-143-3p can be delivered via EVs, allowing them to influence tissue development and regeneration [206, 207]. However, in this context, they exert their effects through different targets other than versican. The deposition of collagen in developing tissues and organs is tightly regulated by the coordinated activity of MMPs and their endogenous inhibitors, the tissue inhibitors of metalloproteinases (TIMPs). During mammary gland development, the miR-212/132 family is expressed specifically in the mammary stroma, where it targets MMP9 [208]. Loss of miR-212/132 leads to upregulation of MMP9, resulting in excessive matrix degradation around developing ducts, disrupted collagen deposition, and impaired ductal morphogenesis. Furthermore, EVs enriched in miR-212/132 have been shown to facilitate differentiation of induced pluripotent stem cells (iPSCs) into pancreatic beta cells [209]. Chondrogenesis, the process by which mesenchymal cells differentiate into chondrocytes and deposit specialized ECMs, involves sequential production of mesenchymal, pre-cartilaginous, and cartilaginous matrices [202]. Among miRNAs implicated in this process, miR-140 regulates cartilage homeostasis by promoting formation and preventing degeneration, with its dysregulation implicated in cartilage-destructive diseases [210]. Exosomes derived from miR-140-5p-overexpressing synovial mesenchymal stem cells promote cartilage regeneration and inhibit osteoarthritis progression in vivo [211]. During fetal development, most of the skeleton is initially composed of cartilage, which is later replaced by bone through the activity of osteoblasts and osteoclasts. This ossification process involves substantial ECM remodeling [212]. Bone ECM differs from that of cartilage in its lower proteoglycan content and in the presence of mineralized collagen, primarily type I and type V collagen. Nephronectin, also known as POEM, is a secreted ECM protein that binds integrin α8β1 and is expressed in various embryonic tissues, including developing long bones in the mouse embryo [202]. miR-378 has been shown to bind the 3’ UTR of nephronectin mRNA, enhancing its expression and promoting osteoblast differentiation [213]. Bone marrow MSC-derived EVs containing miR-378a-5p have demonstrated therapeutic potential to alleviate rheumatoid arthritis [214]. In addition to miR-378, miR-23a also target nephronectin 3’ UTR. EVs enriched with miR-23a-3p, derived from GelMA/nanoclay hydrogels, have been used experimentally to facilitate cartilage regeneration [215]. However, further research is required to elucidate the precise mechanisms through which EV-miRNAs regulate nephronectin expression and osteoblast differentiation.These findings underscore the role of EV-miRNAs in modulating ECM components and remodeling during tissue development (Summarized in Table 4). Nevertheless, important questions remain regarding the spatial and temporal expression patterns of these miRNAs across developmental stages. Moreover, it remains to be clarified whether these miRNAs primarily influence the initiation of differentiation, its maintenance, or both.

**Table 4:** EV-miRNAs regulate ECM remodeling

miRNA	Target gene(s)	ECM regulation	Reference
Multiple	Multiple targets	MBVs from cartilage (anti-angiogenic miRNAs) inhibit endothelial proliferation, migration, and neovessel formation; MBVs from SIS (pro-angiogenic miRNAs) promote angiogenesis, reflecting tissue-specific ECM regulation	[181]
let-7-5p/miR-22-3p/miR-27a-3p/miR-21-5p/miR-23a-3p	TGFBR1/TGFBR2	Inhibit TGF-β signaling by targeting TGF-β receptors to suppress myofibroblast differentiation and excessive ECM deposition	[183]
let-7-5p	TGFBR1	Suppresses fibrosis via TGF-β/Smad signaling; reduces fibroblast activation and collagen I/α-SMA expression	[184]
miR-21-5p/miR-125b-5p	TGFBR2/TGFBR1	Inhibit TGF-β signaling to suppress myofibroblast differentiation and reduce fibrotic ECM deposition	[185]
miR-16/miR-26a/b/miR-141/miR-148a/miR-200a	WNT pathway genes	Inhibit WNT/TGF-β crosstalk and reduce myofibroblast activation and ECM deposition	[186]
miR-17-5p	TGF-β RII	Attenuates osteogenic differentiation of vascular smooth muscle cells under high glucose	[187]
miR-148a-3p	Hsp90b1	Inhibits fibroblast collagen synthesis and secretion by targeting Hsp90b1, suppressing TGF-β1-induced ECM protein expression	[188]
miR-423-5p	COL1A1, α-SMA, Mgll, FAM3	Inhibits hepatic stellate cell differentiation, reducing profibrotic marker expression and ECM protein synthesis	[189]
miR-432-5p	TRAM2	Suppresses collagen biosynthesis to prevent ECM deposition	[190]
miR-206	Rrbp1	Suppresses excessive ECM production by repressing Rrbp1 in fibrogenic cells	[191]
miR-192-5p	IL-17RA	Attenuates hypertrophic scar fibrosis by targeting IL-17RA to regulate Smad signaling, reducing collagen I/III and α-SMA expression	[192]
miR-107	HIF-1α	Antagonizes profibrotic pericyte phenotypes by suppressing a signaling axis (HIF-1α/Notch1/PDGFRβ/YAP1/Twist1), reducing α-SMA and type I collagen expression	[193]
miR-150-5p	MMP14, VEGF	Modulates ECM degradation and pathological angiogenesis by targeting MMP14 and VEGF	[194]
miR-532-5p	RASSF5	Suppresses ECM degradation and fibrosis deposition in nucleus pulposus cells by targeting RASSF5	[195]
miR-202-3p	MMP11	Promotes extracellular matrix formation by targeting MMP11, increasing ECM component levels	[196]
miR-15a	MMP3	Promotes chondrogenic differentiation of nucleus pulposus MSCs by targeting MMP-3, thereby enhancing cartilage matrix formation and reducing ECM degradation	[197]
miR-205-5p	MDM2	Suppresses inflammatory signaling and MMP expression via MDM2 regulation, thereby reducing joint destruction and pathological ECM remodeling in rheumatoid arthritis models	[198]
miR-125a-5p	E2F2	Enhances chondrocyte migration and promotes ECM synthesis by targeting E2F2, increasing collagen II, aggrecan, and SOX9 while reducing MMP-13, thereby attenuating cartilage ECM degradation	[199]
miR-155-5p	Runx2	Enhances ECM secretion in osteoarthritic chondrocytes by targeting Runx2	[200]
miR-199a-5p	Caveolin-1	Promotes muscle fibrosis by inducing fibroblast to myofibroblast conversion, increasing collagen and fibronectin production via reduction of caveolin-1	[201]
miR-138-5p	GPR124	Maintains decidual ECM structure; supports proper tissue remodeling during embryo implantation; prevents inflammation-associated ECM degradation	[206]
miR-212/miR-132	MMP9	Regulates epithelial-stromal interactions and maintains collagen deposition and proper ductal outgrowth	[208]
miR-140-5p	RalA, Wnt5a/Wnt5b	Promotes chondrocyte proliferation and migration while preserving ECM secretion	[211]
miR-378a-5p	IRF1/STAT1 axis	Improves synovial vascular remodeling, indirectly supports ECM integrity and reduces inflammation-related ECM degradation	[214]
miR-23a-3p	PTEN, AKT	Stimulates ECM synthesis and cartilage regeneration	[215]

### EV-miRNA-mediated ECM remodeling during cancer progression

#### EVs as mediators of ECM remodeling within the tumor microenvironment

ECM remodeling is a key driver of tumor progression, significantly influencing cell proliferation, invasion, and metastasis within the tumor microenvironment (TME) [216, 217]. This remodeling is orchestrated by both tumor cells and cancer-associated fibroblasts (CAFs), which secrete ECM components and remodeling enzymes such as lysyl oxidase (LOX) and MMPs. These enzymes increase ECM stiffness and degrade structural barriers, which facilitates immune evasion and tumor spread. Mechanistically, integrin-mediated mechanosignaling and proteolytic activity release growth factors and matrikines from the ECM, thereby enhancing angiogenesis, migration, and proliferation. Hypoxia within the TME further drives this process by inducing the expression of multiple MMPs and vascular endothelial growth factor (VEGF), which together establish pro-angiogenic gradients conducive to vascular sprouting. Additionally, the formation of a dense ECM supports vascular mimicry, enabling tumor cells to adopt endothelial-like characteristics and integrate into the vascular network. As a result, tumor and stromal cells breach the basement membrane via proteolytic degradation or force-mediated mechanisms, often using invadopodia for invasion [218]. CAFs further assist invasion by aligning collagen fibers and forming adhesions with tumor cells to guide their directional migration [219]. Collectively, ECM stiffening and degradation reshape the TME into a pro-tumorigenic landscape, facilitating invasion, angiogenesis, and metastatic dissemination [220].

Among the various drivers of ECM remodeling in cancer, EVs have emerged as multifunctional mediators. EVs secreted by cancer cells facilitate intercellular communication within the TME, serving as vehicles for the transport of biomolecules and signaling cues. Tumor-derived EVs significantly influence cellular behavior, contributing to tumor progression by activating multiple signaling pathways, altering the physiological states of recipient cells, and modulating the TME [43]**.** Tumor cells and cancer-associated fibroblasts (CAFs) secrete EVs abundantly, and these vesicles are enriched with ECM-modifying cargo, including proteases such as MMPs (e.g., MMP9) and membrane-type MMPs (e.g., MT1-MMP) [221]. EVs carrying these enzymes as additional sources contribute to basement membrane degradation, thereby advancing tumor invasion, promote the release of matrikine, and liberate matrix-bound growth factors, thereby advancing tumor invasion. In addition, EVs secreted by cancer cells carry membrane-bound proteases and glycosidases, such as hyaluronidase, which degrade ECM components, including proteins, proteoglycans, and glycoproteins, leading to ECM disassembly and remodeling [222]. Exosome secretion has also been identified as essential for the formation and function of invadopodia. The maturation of invadopodia and their capacity to degrade ECM appear to depend on the delivery of MT1-MMP and potentially other proteinases via exosomes [100]. Furthermore, EVs transport membrane-bound LOX enzymes, which catalyze collagen crosslinking and contribute to ECM stiffening, thereby reinforcing a pro-invasive ECM architecture [222]. In addition to enzymatic cargo, tumor-derived EVs also express surface-bound ADAM (a disintegrin and metalloproteinase) family, which can cleave various cell surface receptors and activate downstream signaling cascades that further support tumor progression [222]. Integrins expressed on the EV membranes selectively bind ECM components like fibronectin or laminin, supporting pre-metastatic niches formation [121]. This interaction also enhances cellular response to matrix stiffness, promoting tumor cell invasion and migration. Importantly, EVs do not function in isolation but coordinate with other components of the TME, including CAFs and immune cells, to sustain a tumor-supportive microenvironment [223]. For example, EVs derived from CAFs have demonstrated potent ECM remodeling capabilities by activating the TGF-β signaling pathway in lung fibroblasts, thereby contributing to the formation of pre-metastatic niches [224]. Recent evidence further highlights the role of MBVs derived from CAFs in shaping tumor-stromal communication [225]. Human mammary CAFs with a myofibroblast phenotype were shown to produce abundant MBVs to transfer plasma-membrane-bound proteins to endothelial cells. These findings underscore that CAF-derived EVs not only remodel the ECM but also directly amplify the pro-tumorigenic crosstalk within the TME.

#### EV-miRNAs as regulators of ECM remodeling in cancer progression

EVs are well recognized for carrying miRNAs between the cells and their role in mediating intercellular communication through the transfer of miRNAs. These EV-associated miRNAs have been implicated in the pathogenesis of numerous diseases, particularly cancer. Within the TME, miRNAs encapsulated in the EVs play a crucial role in cancer progression, notably by enhancing tumor invasiveness and metastatic potential. As summarized in a recent review [36], EV-miRNAs contribute to cancer pathogenesis through different mechanisms. Here, we focus on how EV-miRNAs regulate ECM remodeling during cancer progression.

Tumor-mediated ECM remodeling involves a range of mechanisms, including ECM decomposition, post-translational modification, proteolytic degradation, and force-mediated remodeling [217]. Within this dynamic matrix environment, EV-miRNAs influence ECM structure and function by post-transcriptional and functional alterations in matrix constituents [177]. These changes are essential for initiation of metastasis, as they affect cell proliferation, migration, and invasion. EV-miRNAs can exert direct effects by targeting mRNAs encoding ECM proteins in recipient cells within the TME. For example, in prostate cancer, EV-derived miR-92a-1-5p has been shown to directly target and downregulate COL1A1, reducing type I collagen expression and thereby impacting bone homeostasis [226]. EV-miR-92a released from bone marrow-derived cells enhances the activation of hepatic stellate cells and the expression of ECM proteins, contributing to hepatic pre-metastatic niche formation in lung cancer [227]. Similarly, hypoxic papillary thyroid cancer cells secrete EVs enriched in miR-21-5p, which targets TGFBI and COL4A1 in human umbilical vein endothelial cells (HUVECs), promoting angiogenesis and ECM remodeling [228]. Beyond direct effects, EV-miRNAs also regulate ECM indirectly by modulating the expression of ECM-degrading enzymes such as MMPs. For instance, breast cancer-derived EVs transfer miR-301a-3p, which downregulates TIMP-2 in astrocytes, promoting ECM degradation by removing inhibition of MMP activity [229]. In esophageal squamous cell carcinoma (ESCC), exosomal miR-301a-3p promotes M2 macrophage polarization by inhibiting PTEN and activating the PI3K/AKT pathway, thereby leading to increased secretion of angiogenic and ECM-degrading factors such as VEGFA and MMP9 [230]. Moreover, colorectal cancer cell-derived exosomes have been shown to upregulate the expression of MMP-2, MMP-9, and MMP-11, correlating with increased levels of exosomal miR-21-5p, and its targeting of PDCD4 further supporting the role of EV-miRNAs in proteolytic ECM remodeling [231]. Similarly, miR‑1252‑5p delivered via EVs has been shown to enhance bortezomib sensitivity in multiple myeloma cells by targeting heparanase (HPSE), an enzyme involved in extracellular matrix (ECM) remodeling [232]. In addition, the role of EV-miRNAs in post-transcriptional gene regulation related to ECM remodeling has also been documented. For example, metastatic breast cancer cell-derived EVs contain miR-105, which targets the 3′UTR of ZO-1, a tight junction protein. Downregulation of ZO-1 increases vascular permeability, thereby facilitating metastatic dissemination [233]. A growing number of miRNAs have been implicated in ECM regulation in cancer by modulating the expression of ECM components or their regulators [202, 234], particularly in breast cancer [235]. Some of miRNAs are known to be delivered via EVs to recipient cells [236–238] although the extent to which they directly or indirectly mediate ECM remodeling remains under investigation. Nevertheless, targeting ECM and its cellular receptors via miRNA-mediated pathways delivered by EVs represents a promising strategy for modulating matrix-dependent processes that drive tumor progression [235]. In summary, EV-miRNAs have emerged as important modulators of ECM remodeling in cancer (Summarized in Table 5). By directly targeting ECM constituents or indirectly regulating ECM remodeling proteins, these miRNAs contribute to the dynamic interplay between cancer cells and the ECM, promoting tumor invasion, migration, and metastasis.

**Table 5:** EV-miRNA-mediated regulation of ECM remodeling in cancer

Disease	EV-producing cell	EV-targeted cell	EV-miRNA	miRNA target gene	ECM/tissue effect	Reference
Multiple Myeloma	Hypoxia-resistant MM cells	HUVECs	miR-135b	FIH-1	Enhanced angiogenesis and neovascular ECM remodeling in the hypoxic bone marrow microenvironment	[92]
Hepatocellular Carcinoma	Hypoxic HCC cells	HUVECs	miR-3174	HIPK3	Increased pathological angiogenesis with enhanced vascular permeability and neovascular ECM remodeling	[96]
Neuroblastoma	Neuroblastoma cells, monocytes/macrophages	Monocytes/Neuroblastoma cells	miR-21, miR-155	TRL8, TERF1	Altered tumor-associated immune microenvironment	[147]
Prostate Cancer (Bone metastasis)	Osteogenic tumor cells	Osteoclast precursors/bone stromal cells	miR-92a-1-5p	COL1A1	Osteoclast activation and degradation of collagen I–rich bone ECM	[226]
Lung Cancer (Pre-metastatic niche)	Bone marrow–derived cells	Hepatic stellate cells	miR-92a-3p	SMAD7	TGF-β activation and fibrotic ECM deposition in liver niche	[227]
Papillary Thyroid Cancer	Cancer cells	HUVECs	miR-21-5p	TGFBI, COL4A1	Increased angiogenesis and basement membrane ECM remodeling	[228]
Breast Cancer (Brain metastasis)	Breast cancer cells	Astrocytes	miR-301a-3p	TIMP-2	Increased MMP activity and ECM degradation at blood–brain barrier	[229]
Esophageal Squamous Cell Carcinoma	ESCC cells	Macrophages	miR-301a-3p	PTEN	M2 polarization, angiogenesis, and ECM degradation via PI3K/AKT	[230]
Colorectal Cancer	Colon cancer cells	Cancer cells (autocrine)	miR-21-5p	PDCD4	Upregulation of MMP-2, MMP-9, MMP-11 and ECM degradation	[231]
Multiple Myeloma	HEK293T engineered EVs	MM cells (U266, RPMI-8226)	miR-1252-5p	HPSE	Reduced heparan sulfate degradation; decreased ECM remodeling	[232]
Breast Cancer (Metastatic)	Metastatic cancer cells	HMVECs	miR-105	ZO-1	Endothelial tight-junction disruption and vascular barrier breakdown	[233]
Pancreatic Cancer	Pancreatic cancer cells (PaC)	Stromal fibroblasts	miR-155	TP53INP1	Promotion of fibroblast reprogramming into CAFs, contributing to ECM remodeling	[236]
Melanoma	Acid-adapted melanoma cells	Macrophages/Endothelial cells	miR-214	Not directly identified	Enhanced vascular permeability and ECM remodeling-associated trans-endothelial migration	[237]
Glioblastoma	Hypoxic GBM cells	Endothelial/stromal cells	miR-210-3p	EFNA3	Enhanced ECM remodeling with increased angiogenesis and vascular permeability	[238]

## Conclusion

The intricate and reciprocal relationship between the ECM and EV-miRNAs forms a dynamic regulatory axis that governs tissue homeostasis, development, and disease progression. Once considered primarily a structural scaffold, the ECM is now recognized as a critical modulator of EV biogenesis, cargo sorting, mobility, and uptake, thereby influencing the functional delivery of EV-miRNAs. In parallel, EV-miRNAs contribute to ECM remodeling by modulating gene expression profiles that dictate the synthesis, degradation, and organization of matrix components. This bidirectional communication is not only essential for normal physiological processes such as tissue morphogenesis and repair but is also exploited in pathological contexts, including tumor progression, where aberrant ECM–EV-miRNA signaling reinforces disease progression.

Despite significant advances in characterizing the molecular players and mechanisms underpinning this interplay, substantial gaps remain in our understanding of the spatio-temporal regulation, cell-type specificity, and functional thresholds required for EV-miRNAs to effect meaningful biological change. Deciphering the contextual dependencies, such as matrix stiffness and receptor-ligand interactions that modulate EV-mediated miRNA transfer, will be pivotal in leveraging this axis for therapeutic intervention. Future research should prioritize integrated in vivo models, quantitative assessments of EV-miRNA stoichiometry, and the development of technologies to track EV fate and cargo delivery with precision.

Overall, the ECM and EV-miRNA interplay represents a promising frontier in regenerative biology and disease therapeutics. A deeper mechanistic understanding of this crosstalk will not only elucidate fundamental aspects of intercellular communication but also inform the rational design of ECM- and EV-based strategies for targeted tissue modulation and clinical translation.

## Data Availability

Data sharing is not applicable to this article as no datasets were generated or analyzed during the current study.
